# Joint association of METS-IR and uric acid with stoke, mediated by C-reactive protein

**DOI:** 10.3389/fendo.2024.1448021

**Published:** 2024-11-20

**Authors:** Shan Jiang, Xinyi Zhang, Chengning Song, Guangfu Wu, Aicheng Yang

**Affiliations:** ^1^ Nephropathy Center, The Alliated Jiangmen Traditional Chinese Medicine (TCM) Hospital of Jinan University, Jiangmen, China; ^2^ College of Traditional Chinese Medicine, Tianjin University of Chinese Medicine, Tianjin, China

**Keywords:** CHARLS, METS-IR, UA, stroke, joint effect

## Abstract

**Objectives:**

To explore the dose-response relationship between the Metabolic Score of Insulin Resistance (METS-IR), uric acid (UA) and the risk of stroke incidence, the mediating role of C-reactive protein (CRP) in the above relationship, as well as the joint effect of METS-IR and UA on the risk of stroke incidence.

**Methods:**

Participants from the CHARLS study were included in this cohort study. Logistic regression models were used to estimate the odds ratios (ORs) and corresponding 95% confidence intervals (CIs) for the associations of METS-IR and UA with the risk of incident stroke. The dose-response relationships of METS-IR and UA with stroke risk were assessed by restricted cubic spline regression. The mediation models were employed to estimate the potential mediating effects of CRP on the associations of METS-IR and UA with stroke risk. Logistic regression analysis was carried out to analyse the association of stroke and MRTS-IR combined with UA.

**Result:**

During a 9-year follow-up from 2011 to 2018, 570 incident cases of stroke were documented among 7,343 total participants. Per interquartile range increases in METS-IR and UA were associated with the increased risk of incident stroke, with the OR (95% CI) of 1.61 (1.44, 1.80) and 1.18 (1.05, 1.32) respectively. A dose-response function showed that METS-IR had a nonlinear relationship (*P* for nonlinear=0.047) and UA had a linear relationship (*P* for nonlinear=0.247) with the stroke risk. CRP had significant mediated effects on the associations of METS-IR and UA with stroke risk, and the proportion of mediation was 9.01% and 26.34% respectively (all *P* < 0.05). The results of joint effect showed that participants with high levels of METS-IR and UA had the highest increased risk of stroke compared to the participants with low levels of METS-IR and UA.

**Conclusion:**

METS-IR and UA levels were positively associated with an increased risk of stroke onset, and CRP mediated these relationships. Improving insulin sensitivity and regulating CRP and uric acid levels may be important for preventing the risk of stroke occurrence.

## 1 Introduction

Stroke, also known as cerebrovascular accident, is a leading cause of death and disability worldwide (1, 2). According to the Global Burden of Disease Study, stroke is responsible for significant mortality, morbidity, and long-term disability, particularly in low- and middle-income countries (3). In 2019, there were over 100 million patients who had a stroke and 12 million new stroke cases globally (4). The average age of stroke patients in China is 66.4 years (5), almost 10 years younger than white Europeans (6), leading to a significant reduction in life expectancy among the working-age population. As the world’s most populous and rapidly ageing population, China faces an increasing challenge in reducing stroke morbidity and mortality.

A growing number of studies have confirmed that insulin resistance (IR) is an independent risk factor for cerebrovascular disease (7, 8). Insulin resistance can accelerate thrombosis, cause endothelial cell dysfunction, and promote the development of atherosclerosis, which in turn leads to stroke. Meanwhile, IR is also an important cause of poor prognosis in ischaemic stroke, which can exacerbate neurological deterioration during hospitalisation and trigger stroke recurrence (9, 10). Therefore, IR is considered a key risk factor for stroke development. The Metabolic Score of Insulin Resistance (METS-IR) is a more accurate measure of insulin sensitivity by combining fasting blood glucose (FBG), fasting triglycerides (TG), body mass index (BMI), and high-density lipoprotein cholesterol (HDL-C) (11). It is used as a reliable predictor of cardiovascular events, hypertension, diabetes mellitus, and other multi-system diseases (12–14). In addition, the relationship between blood uric acid (UA) levels and stroke remains inconclusive (15, 16). Elevated UA levels may contribute to stroke by promoting oxidative stress, endothelial dysfunction, and inflammation, all of which are known to play a role in cerebrovascular events (17). Moreover, the combined impact of UA and IR on stroke risk is not yet fully understood. Given the prevalence of both hyperuricemia and insulin resistance in at-risk populations, it is crucial to further investigate how these factors interact.

Therefore, the present study constructs a 9-year prospective cohort study based on the China Health and Retirement Longitudinal Studies (CHARLS) database to explore the dose-response relationship between METS-IR, UA, and the risk of stroke incidence. Additionally, we examined the joint effect of METS-IR and UA on stroke risk. Moreover, since both METS-IR and UA can induce inflammatory responses, we selected C-reactive protein (CRP) as a mediator to investigate whether inflammation plays a role in mediating the relationship between these factors and stroke, providing a clearer understanding of the underlying mechanisms contributing to stroke risk.

## 2 Methods

### 2.1 Study design and population

The China Health and Retirement Longitudinal Study (CHARLS) is a national prospective survey conducted by the China Center for Economic Research at Peking University. The database collects diverse and high-quality data among middle-aged and older Chinese individuals (age ≥ 45 years). The study design and methods have been reported in detail previously (18). In brief, the Ethical Review Committee at Peking University granted approval to CHARLS (IRB00001052-11,015) in 2008. The CHARLS enrolled over 17,000 individuals from approximately 10,000 households in 150 county-level units and 450 village-level units at baseline in 2011. All participants completed an informed consent form and were revisited every two to three years, with four follow-up surveys having been conducted thus far. CHARLS employed a robust multistage, stratified sampling approach to ensure representativeness. The sampling units were selected from diverse regions across China, and participants were chosen randomly within these regions. The survey uses a face-to-face, computer-assisted personal interviewing (CAPI) method to gather data, ensuring both accuracy and depth in the responses (19).

**Figure 1:**
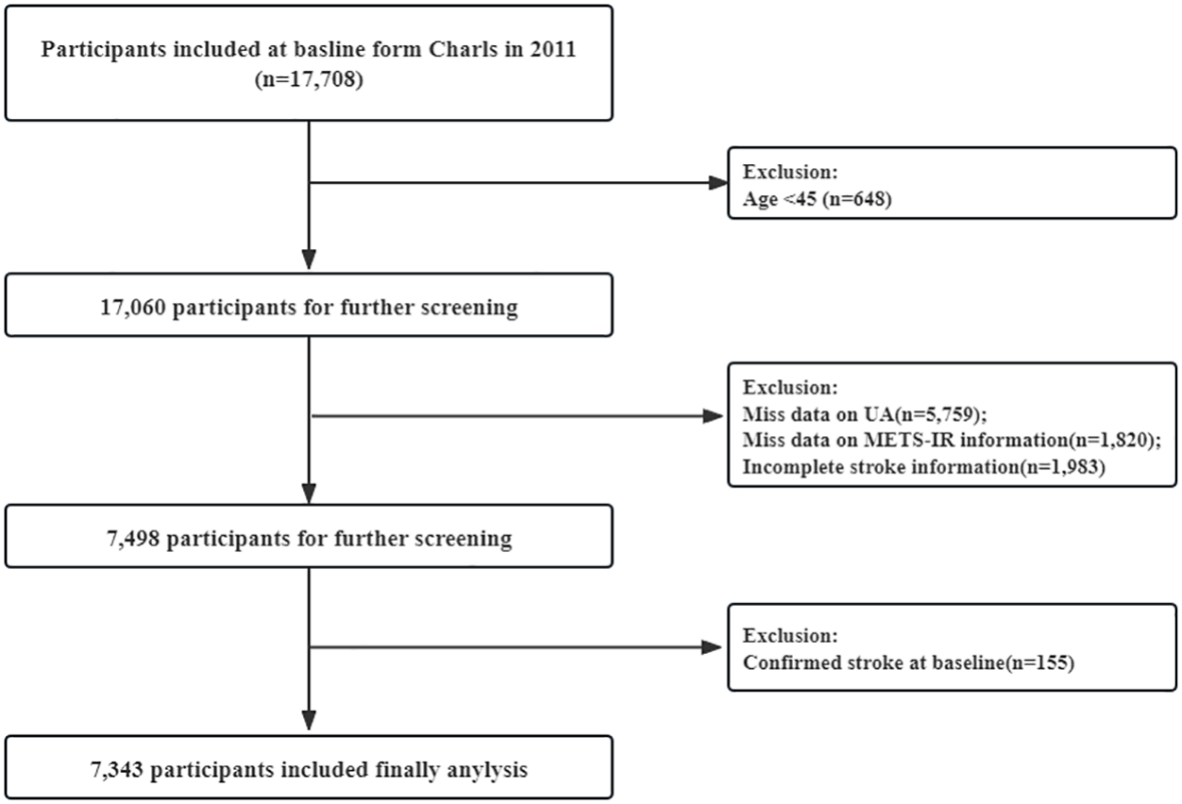
Flow chart for the selection of participants in the cohort study from Charls from 2011 to 2020 (n=7,343).

In current analyses, among the 17,708 participants initially screened, 10,365 were excluded because of incomplete age information or age <45 years (648), UA (5,759), and incomplete METS-IR information (1,820), incomplete stroke information (1,983), or confirmed stroke at baseline (155). Finally, a total of 7,343 participants were included in the analysis (**Figure 1**).

### 2.2 Assessment of METS-IR

Data collection for CHARLS involved venous blood assays and a physical examination. During the examination, the doctor conducted anthropometric measurements and calculated the body mass index (BMI). Professional medical staff collected fasting blood samples from the participants. Fasting plasma glucose (FPG), total cholesterol (TC), high-density lipoprotein cholesterol (HDL-C), and triglyceride (TG) levels were determined using enzymatic colorimetric tests. Serum uric acid (SUA) levels were measured using the UA Plus method. METS-IR calculation formula: Ln [(2 × FBG (mg/dL)) + TG (mg/dL)] × BMI (kg/m2))/(Ln [HDL-C (mg/dL)]) (20).

### 2.3 Outcome ascertainment

In accordance with established precedents (21), stroke was diagnosed by professionals and the information was collected by a questionnaire survey. Briefly, participants were asked, “Have you been diagnosed with stroke?” Those who responded affirmatively were identified as stroke occurrences and were enrolled in the study. Participants were followed from assessment in 2011 until the occurrence of stroke or the most recent survey in 2020, whichever came first.

### 2.4 Covariates

Sociodemographic and lifestyle covariates were assessed by the baseline questionnaire or measurements. According to previous studies (22–24), demographic characteristics were essential covariates because they are major risk factors for stroke, with well-documented differences in incidence and outcomes. Education and consumption level were included as proxies for socioeconomic status, both linked to stroke risk. Smoking and alcohol consumption were also considered, as they are well-known modifiable risk factors contributing to vascular damage. Hence, the current analyses adjusted covariates for age (continuous), sex (male or female), education (primary school and below, high school, college or above), marital status (married, never married, separated, or widowed), location (urban or rural), consumption level (continuous), smoking status (never, former, and current), and drinking alcohol status (never, former, and current).

### 2.5 Statistical analysis

All analyses were performed with R (version 4.2.2). Chi-square tests (categorical variables), rank-sum tests (continuous variables without normal distribution), or Student t-test (continuous variables with normal distribution) were used to assess participants’ demographic characteristics across different stroke status in the CHARLS. Continuous variables were presented as median (interquartile range (IQR)) or mean ± standard deviation (SD) and categorical variables as number (%).

METS-IR and UA were used as a continuous variable (per IQR increase) or categorised into four quartiles (Q1, Q2, Q3, and Q4) (24, 25). Logistic regression models were used to estimate the odds ratios (ORs) and corresponding 95% confidence intervals (CIs) for the associations of METS-IR and UA with the risk of incident stroke. Three models were used in this study: model 1 (not adjusted for any covariates), model 2 (adjusted for age, sex, education, marital status, and location), model 3 (further adjusted for consumption level, smoking status, and drinking alcohol status). Missing data for covariates were filled in using multiple interpolation. The trend test across increasing exposure groups was calculated using integer values (1, 2, 3, and 4). The dose-response relationships of METS-IR and UA with stroke risk were assessed by restricted cubic spline regression with three knots placed at the 25th, 50th, and 75th percentile.

The mediation models were employed to estimate the potential mediating effects of CRP on the associations of METS-IR and UA with stroke risk by using the “mediation” package in R (26, 27). The mediation analyses utilised bootstrapped procedures with 1000 simulations. The direct effect indicated the impact of METS-IR and UA on stroke without a mediator, while the indirect effect denoted the influence of METS-IR and UA on stroke through the mediator. The proportion of mediation was calculated by dividing IE by TE (total effect).

The joint effects of METS-IR and UA on stroke were also estimated. We classified METS-IR and UA into high and low levels by greater than (or equal to) or below the median. Participants were then divided into four groups based on the levels of above two variables: high METS-IR and high UA, high METS-IR and low UA, low METS-IR and high UA, and low METS-IR and low UA. Individuals with low levels of UA and low levels of METS-IR as the references, and logistic regression model was applied to analyse the association of combined effects of METS-IR and UA with stroke risk.

## 3 Results

### 3.1 Baseline characteristics of the study population

The mean age of study participants was 58.16 ± 8.48 years and 55.67% were female. During a 9-year follow-up from 2011 to 2018, 570 incident cases of stroke were documented among 7,343 total participants. The demographic characteristics of the study participants with or without stroke were listed in **Table 1**. Overall, stroke patients were more likely to be older and smokers, and had higher BMI, blood glucose, total cholesterol, total triglycerides, low density lipoprotein cholesterol, and CRP as well as lower levels of high density lipoprotein cholesterol compared with non-stroke participants (*P* < 0.05). Besides, participants with stroke had higher levels of UA (median: 4.37 vs. 4.24) and METS-IR (median: 36.94 vs. 34.08) compared to non-stroke participants. Similarly, in the stroke group, a higher proportion of participants are in the highest quartiles of METS-IR (35.09%) and UA (31.40%) compared to the non-stroke group (24.15% and 24.45%, respectively).

**Table 1:** Baseline characteristics of study population by stroke status at follow-up.

Variables	Total (n = 7343)	Non-stroke (n = 6773)	Stroke (n = 570)	Statistic	*P*
Age	58.16 ± 8.48	57.97 ± 8.50	60.44 ± 7.92	t=-7.12	<.001
Sex				χ²=1.17	0.279
Female	4088 (55.67)	3783 (55.85)	305 (53.51)		
Male	3255 (44.33)	2990 (44.15)	265 (46.49)		
Education				χ²=2.64	0.451
Below Primary School	3470 (47.26)	3197 (47.20)	273 (47.89)		
Primary School	1606 (21.87)	1471 (21.72)	135 (23.68)		
Junior High School	1525 (20.77)	1412 (20.85)	113 (19.82)		
High School and above	742 (10.10)	693 (10.23)	49 (8.60)		
Marital				χ²=1.15	0.284
Married	742 (10.10)	677 (10.00)	65 (11.40)		
Non-Married	6601 (89.90)	6096 (90.00)	505 (88.60)		
Location				χ²=0.92	0.339
Urban	2426 (33.04)	2248 (33.19)	178 (31.23)		
Rural	4917 (66.96)	4525 (66.81)	392 (68.77)		
History of Alcohol Consumption				χ²=3.34	0.068
No	4552 (61.99)	4219 (62.29)	333 (58.42)		
Yes	2791 (38.01)	2554 (37.71)	237 (41.58)		
Current Alcohol Consumption				χ²=0.03	0.864
No	4936 (67.22)	4551 (67.19)	385 (67.54)		
Yes	2407 (32.78)	2222 (32.81)	185 (32.46)		
History of Smoking				χ²=6.64	0.01
No	4619 (62.90)	4289 (63.32)	330 (57.89)		
Yes	2724 (37.10)	2484 (36.68)	240 (42.11)		
Current Smoking Status				χ²=1.66	0.198
No	5172 (70.43)	4784 (70.63)	388 (68.07)		
Yes	2171 (29.57)	1989 (29.37)	182 (31.93)		
Per Capita Household Consumption	4770.00 (2800.00, 8209.07)	4777.00 (2788.00, 8228.00)	4621.00 (2884.50, 8120.00)	Z=-0.03	0.975
BMI(kg/m²)	23.22 (20.99, 25.84)	23.16 (20.91, 25.73)	24.14 (21.85, 26.85)	Z=-6.04	<.001
Glucose (mg/dl)	102.24 (94.32, 112.14)	102.06 (94.14, 111.96)	103.77 (95.99, 115.38)	Z=-3.61	<.001
TC (mg/dl)	190.98 (168.17, 215.72)	190.98 (168.17, 215.34)	194.07 (171.26, 221.43)	Z=-2.53	0.012
TG (mg/dl)	105.32 (75.22, 147.79)	104.43 (74.34, 147.79)	115.05 (85.85, 154.65)	Z=-4.85	<.001
HDL-C (mg/dl)	49.48 (40.59, 59.92)	49.87 (40.59, 60.31)	46.78 (39.43, 55.67)	Z=-5.02	<.001
LDL-C (mg/dl)	115.21 (94.72, 137.63)	114.82 (94.72, 137.24)	119.27 (95.30, 141.11)	Z=-2.37	0.018
CRP (mg/dl)	0.94 (0.52, 1.85)	0.91 (0.51, 1.80)	1.34 (0.69, 2.43)	Z=-7.74	<.001
UA (mg/dl)	4.25 (3.55, 5.10)	4.24 (3.55, 5.08)	4.37 (3.59, 5.37)	Z=-2.86	0.004
METS-IR	34.26 (29.78, 39.95)	34.08 (29.64, 39.70)	36.91 (31.74, 41.91)	Z=-7.10	<.001
METS-IR quantile				χ²=46.60	<.001
Q1(<29.78)	1836 (25.00)	1746 (25.78)	90 (15.79)		
Q2(29.78-34.26)	1836 (25.00)	1701 (25.11)	135 (23.68)		
Q3(34.26-39.95)	1835 (24.99)	1690 (24.95)	145 (25.44)		
Q4(>39.95)	1836 (25.00)	1636 (24.15)	200 (35.09)		
UA quantile				χ²=13.68	0.003
Q1(<3.55)	1840 (25.06)	1708 (25.22)	132 (23.16)		
Q2(3.55-4.25)	1834 (24.98)	1702 (25.13)	132 (23.16)		
Q3(4.25-5.09)	1834 (24.98)	1707 (25.20)	127 (22.28)		
Q4(>5.09)	1835 (24.99)	1656 (24.45)	179 (31.40)		

*p*-Values were calculated from chi-square tests (categorical variables) or rank-sum tests (continuous variables without normal distribution), or t-tests (continuous variables with normaldistribution).

BMI, Body Mass Index; Glucose, Blood Glucose; TC, Total Cholesterol; TG, Triglyceride; HDL-C, High-density Lipoprotein Cholesterol; LDL-C, Low-density Lipoprotein Cholesterol; CRP, C-reactive Protein; UA, Uric Acid; METS-IR, Metabolic Score for Insulin Resistance.

### 3.2 Association of UA and METS-IR with the risk of incident stroke

**Figure 2:**
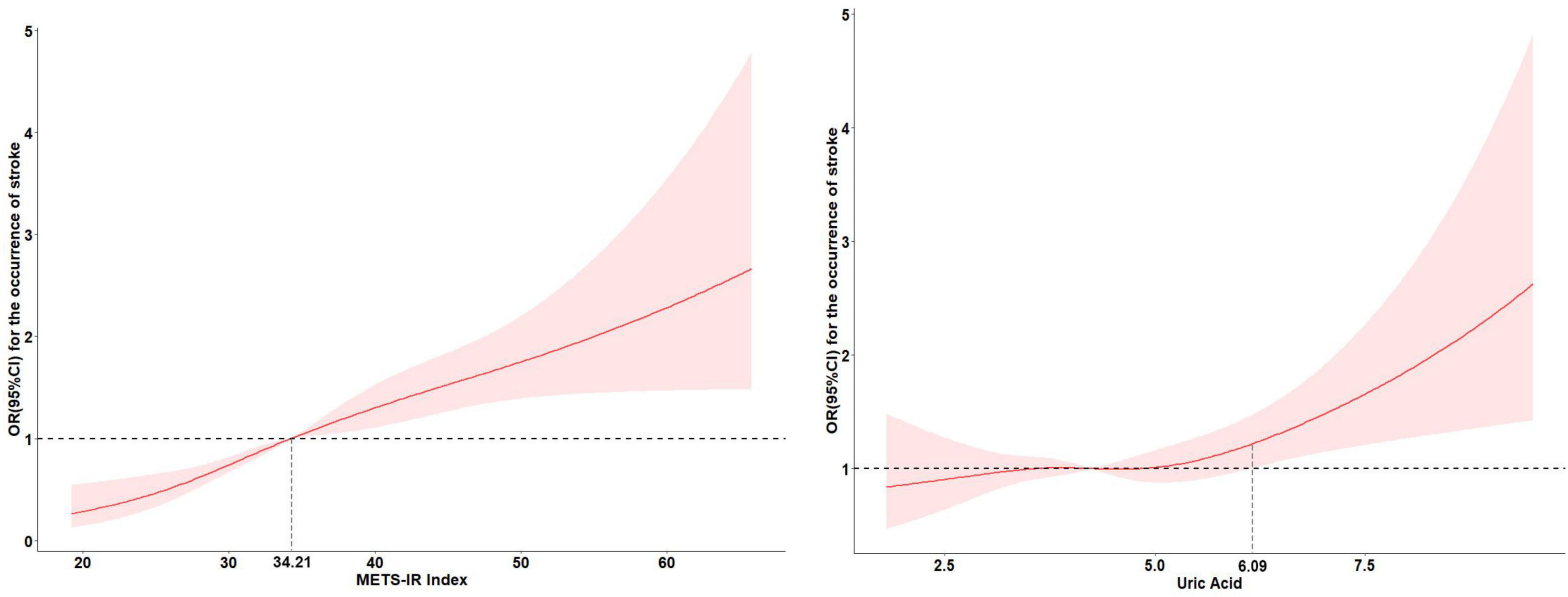
Adjusted cubic spline models of the association between UA and METS-IR and new-onset stroke.

**Table 2** displayed the associations of UA and METS-IR with the stroke risk by logistic regression models. In unadjusted models, per IQR increases in METS-IR and UA were associated with the increased risk of incident stroke, with the OR (95% CI) of 1.48 (1.33, 1.65) and 1.21 (1.09, 1.34) respectively. Model 2 showed that per IQR increases in METS-IR and UA increased stroke risk by 61% and 18% respectively [OR (95% CI): 1.61 (1.44, 1.79) and 1.18 (1.05, 1.33)]. Above findings were consistent with the results of quantile analyses (all *P* for trend < 0.05). In the fully adjusted models, per IQR increases in METS-IR and UA were associated with the increased risk of incident stroke, with the OR (95% CI) of 1.61 (1.44, 1.80) and 1.18 (1.05, 1.32) respectively. Compared with the lowest levels (Q1), the highest exposure quantile (Q4) of METS-IR [OR (95% CI): 2.87 (2.20, 3.76)] and UA [OR (95% CI): 1.30 (1.00, 1.68)] increased the incident risk of stroke. A dose-response function showed that METS-IR had a nonlinear relationship (*P* for nonlinear=0.047) and UA had a linear relationship (*P* for nonlinear=0.247) with the incident stroke risk (**Figure 2**).

**Table 2:** Associations between baseline METS-IR and UA with follow-up incident stroke.

	Model 1	*P*	Model 2	*P*	Model 3	*P*
METS-IR per IQR	1.478(1.328,1.645)	<0.001	1.605(1.437,1.791)	<0.001	1.606(1.437,1.795)	<0.001
Q1(<29.78)	ref		ref		ref	
Q2(29.78-34.26)	1.540 [1.172, 2.032]	0.002	1.702 [1.292, 2.252]	<0.001	1.704 [1.293, 2.256]	<0.001
Q3(34.26-39.95)	1.664 [1.272, 2.190]	<0.001	1.913 [1.455, 2.528]	<0.001	1.921 [1.459, 2.541]	<0.001
Q4(>39.95)	2.372 [1.839, 3.081]	<0.001	2.851 [2.192, 3.733]	<0.001	2.865 [2.198, 3.760]	<0.001
*P* for trend	<0.001		<0.001		<0.001	
UA per IQR	1.205(1.086,1.337)	0.000	1.182(1.054,1.325)	0.004	1.176(1.048,1.319)	0.006
Q1(<3.55)	ref		ref		ref	
Q2(3.55-4.25)	1.004 [0.781, 1.289]	0.978	0.977 [0.758, 1.259]	0.858	0.987 [0.766, 1.273]	0.921
Q3(4.25-5.09)	0.963 [0.747, 1.240]	0.768	0.925 [0.711, 1.202]	0.559	0.920 [0.707, 1.196]	0.532
Q4(>5.09)	1.399 [1.107, 1.771]	0.005	1.312 [1.016, 1.697]	0.038	1.295 [1.002, 1.677]	0.049
*P* for trend	0.008		0.050		0.069	

Model 1 was crude model. Model 2 was adjusted for age, gender, education level, location, and marital status. Model 3 was furthrt for smoking status, drinking status and Per Capita Household.

METS-IR, Metabolic Score for Insulin Resistance; UA, Uric Acid (mg/dl); IQR, interquartile range.

### 3.3 Mediation analysis

Furthermore, mediation analyses were performed to evaluate the potential mediation effects of CRP on the associations of METS-IR and UA with the risk of stroke. As shown in **Table 3**, CRP had significant mediated effects on the associations of METS-IR and UA with stroke risk, and the proportion of mediation was 9.01% and 26.34% respectively (all *P* < 0.05).

**Table 3:** Mediation of the association between METS-IR and UA with Stroke in Charls by CRP.

Variable		Estimate	95%CI Lower	95%CI Upper	*P*-value
(METS-IR)-(CRP)-Stroke					
	ACME (average)	0.000072	0.000035	0.000001	<0.001 ***
	ADE (average)	0.000676	0.000586	0.000001	<0.001 ***
	Prop. Mediated	0.090100	0.055200	0.170000	<0.001 ***
(UA)-(CRP)-Stroke					
	ACME (average)	0.001327	0.000835	0.000001	<0.001 ***
	ADE (average)	0.003615	0.001127	0.010000	0.040*
	Prop. Mediated	0.263440	0.123045	0.590000	0.040*

CI, confidence interval; METS-IR, Metabolic score for insulin resistance; UA, Uric Acid (mg/dl); CRP, C-reactive Protein (mg/dl); IQR, interquartile range; ADE, Average Direct Effect; ACME, Average Causal Mediation Effect. * means p < 0.05, and *** means p < 0.001.

### 3.4 Joint effect of UA and METS-IR on the risk of incident stroke

**Figure 3:**
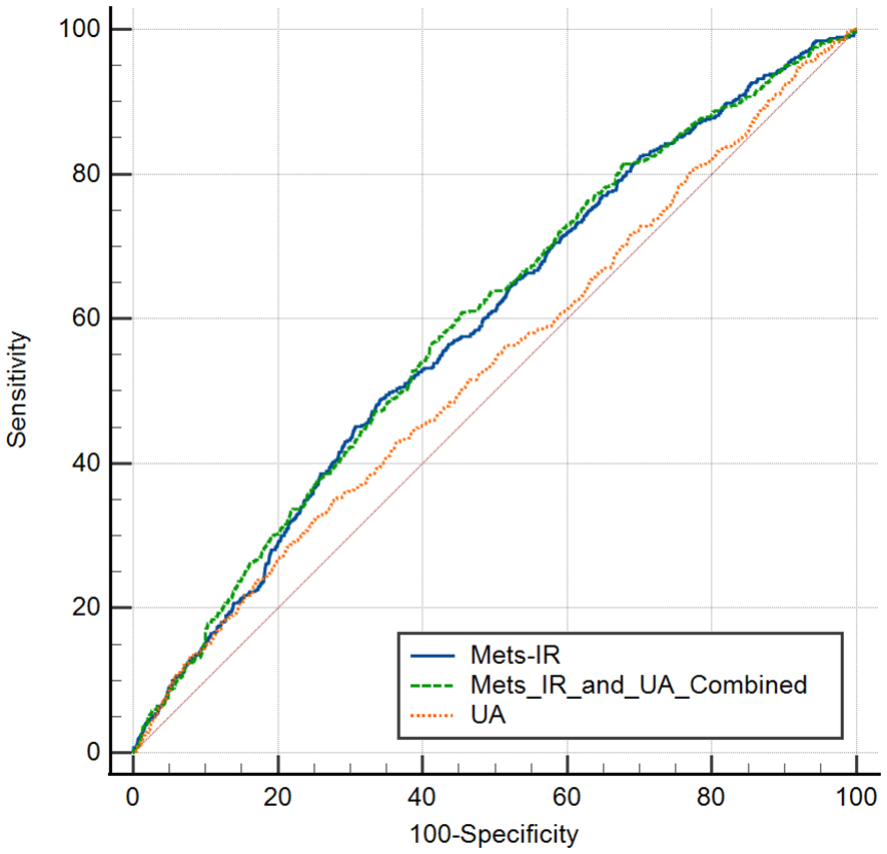
ROC curves for pedicting stroke risk using Mets-IR, uric acid (UA), and their combination.

We next analysed the joint effects of METS-IR and UA on stroke risk. Compared with the participants with low levels of METS-IR and UA, those with high levels of METS-IR but low levels of UA had the increased risk of stroke [OR (95% CI): model 1: 1.61 (1.25, 2.08); model 2: 1.77 (1.37, 2.29); model 3: 1.76 (1.36, 2.28)] (all *P* < 0.001), while those with low levels of METS-IR but high levels of UA had no significance in the risk of stroke [OR (95% CI): model 1: 1.17 (0.89, 1.53); model 2: 1.05 (0.79, 1.39); model 3: 1.03 (0.78, 1.37)]. Moreover, compared to the participants with low levels of METS-IR and UA, participants with high levels of METS-IR and UA had the highest increased risk of stroke [OR (95% CI): model 1: 1.78 (1.41, 2.26); model 2: 1.82 (1.42, 2.33); model 3: 1.79 (1.40, 2.30)] (all *P* < 0.001) (**Table 4**). We have plotted the ROC curves and performed a pairwise comparison of the predictive performance for METS-IR, UA, and their combined effect (**Figure 3**). As shown in the results, the area under the curve (AUC) for the combined METS-IR and UA model (0.595) is higher compared to METS-IR (0.589) or alone (0.536). The combination of METS-IR and UA shows a statistically significant improvement in predictive performance over UA alone (*P* < 0.0001).

**Table 4:** Association of combined METS-IR and UA analysis with stroke.

	Model 1	*P*	Model 2	*P*	Model 3	*P*
Low UA/Low METS-IR	ref		ref		ref	
Low UA/High METS-IR	1.61 [1.25, 2.08]	<0.001	1.77 [1.37, 2.29]	<0.001	1.76 [1.36, 2.28]	<0.001
High UA/Low METS-IR	1.17 [0.89, 1.53]	0.266	1.05 [0.79, 1.39]	0.734	1.03 [0.78, 1.37]	0.837
High UA/High METS-IR	1.78 [1.41, 2.26]	<0.001	1.82 [1.42, 2.33]	<0.001	1.79 [1.40, 2.30]	<0.001

Model 1 was crude model. Model 2 was adjusted for age, gender, education level, location, and marital status. Model 3 was further for smoking status, drinking status and per capita household.

METS-IR, Metabolic Score for Insulin Resistance; UA, Uric Acid (mg/dl); IQR, interquartile range.

## 4 Discussion

To the best of our limited knowledge, this is the first prospective cohort study to jointly explore METS-IR and UA levels with the risk of stroke onset, and to analyse the mediating role of CRP in the above relationships. We found that both METS-IR and serum UA levels were positively correlated with the risk of stroke onset. The dose-response curves indicated a nonlinear relationship between METS-IR and the risk of stroke onset, whereas there was a linear relationship between blood uric acid levels and the risk of stroke onset. CRP had a significant mediating role in the association of METS-IR and UA with stroke risk. The results of joint effects suggested that patients with both high METS-IR and high uric acid (UA) levels had the highest risk of stroke. This study provided a reliable reference for the early detection of people at high risk of stroke and the treatment of the disease.

With the aggravation of population aging, the proportion of potentially high-risk groups for stroke in China has been expanding. Study of stroke risk factors is important for the early identification of high-risk groups and improvement of prognosis. It is well known that IR is one of the important risk factors for stroke (28, 29). IR can cause NO reduction and vasoconstrictor endothelin-1 (ET-1) increase involving in the phosphatidylinositol 3-kinase (PI3K) and mitogen-activated protein kinase (MAPK) signalling pathways, which in turn impairs endothelial cell function and enhances platelet adhesion, activation and aggregation, leading to thrombosis and increased stroke risk (30, 31). METS-IR is a novel index to assess insulin resistance, and we found that METS-IR levels were positively associated with stroke risk, which is consistent with previous findings. For example, Cai et al. (32) found that patients with the highest quartile of METS-IR exhibited a higher risk of stroke (HR, 1.80; 95% CI, 1.50-2.17) compared to the lowest quartile group, through a cohort study of up to 4.8 years. We had a follow-up of up to 9 years, further confirming that METS-IR levels can be used as a reliable predictor of stroke risk. We also found that UA levels were strongly associated with stroke risk, with a 17.6% increase in stroke risk for each IQR increase in UA. This is in line with the results of Khalil et al. who found that for every one unit increase in UA levels, the odds of ischaemic stroke increased by 25% (15). Mechanistically, high UA levels may induce inflammatory responses and oxidative stress by disrupting the integrity of the vascular endothelium and promoting the expression of nod-like receptor family pyrin domain-containing 3 (NLRP3) inflammasome vesicles; as well as decreasing nitric oxide bioavailability and disrupting vascular functions and other factors that contribute to the development of stroke (33, 34). In addition, hyperuricaemia may also increase the risk of stroke by promoting the development of diseases such as atherosclerosis, hypertension and gout (35–37).

In addition, the mediating effect results showed that CRP could act as a mediator between METS-IR and stroke risk, and UA and stroke risk, with mediation ratios of 9.01% and 26.34%, respectively. This can be explained by the fact that METS-IR and UA levels can directly affect the risk of stroke occurrence and can also indirectly affect the risk of stroke by affecting CRP levels. Shahid et al. found that CRP levels were increasing with the increase of IR (38). Minato-Inokawa et al. also found that CRP levels were also higher in women with the presence of IR (39). Mendelian studies have shown a significant causal relationship between CRP and hypertensive heart disease, which is a significant risk factor for stroke (40). The results of meta-analysis based on 3893 participants showed that CRP concentrations were significantly higher in patients with cognitive decline after stroke compared to non-cognitive decline patients (41). Thus, METS-IR levels may indirectly affect the risk of stroke occurrence by influencing CRP levels, and this effect may extend to changes in cognitive function after stroke. Similarly, UA is considered to be a product of oxidative stress, and increased levels can activate inflammatory pathways leading to the release of inflammatory factors such as CRP and TNF-α (42). And increased levels of CRP can promote stroke risk (43). Therefore, the results of mediation analyses of CRP as an inflammatory marker suggest that METS-IR and UA levels may affect stroke risk by influencing the level of inflammation and degree of oxidative stress. This suggests that lowering the level of inflammation is one of the effective treatments to improve stroke risk.

Meanwhile, the results of the joint analysis revealed that stroke risk was highest in those with high levels of both METS-IR and UA. This suggests that insulin resistance and hyperuricemic status may be interrelated in multiple physiological and pathological processes that together influence stroke risk. This suggests that individualised therapeutic strategies for stroke prevention may be required for clinicians dealing with different individuals. For those with both insulin resistance and high uric acid levels, a combination of therapeutic measures, including glycemic control, lipid regulation, and control of uric acid levels, may need to be considered to reduce the risk of stroke.

This study has certain strengths, firstly, to our limited knowledge, this is the first study to explore the relationship between the combined effect of METS-IR and UA levels on the risk of stroke development, which at some level illustrates the role of insulin resistance and inflammation levels in the development of stroke. Second, this is a nationwide large-sample, prospective cohort study with a high level of evidence-based evidence, which provides a reliable basis for the causal relationship between METS-IR and UA levels and the risk of stroke development, and provides a certain reference for early screening and the treatment of stroke. Furthermore, we performed a dose-response relationship to quantify and visualise the relationship between METS-IR and UA levels and the risk of stroke onset. Finally, we performed a mediation analysis using CRP as a mediator, providing an explanatory perspective on the relationship between METS-IR and uric acid (UA) levels and stroke risk.

However, our study also has some limitations. First, the diagnosis of stoke was based on self-report, which, although studies have shown it to be relatively reliable, does not completely prevent the occurrence of erroneous reports (44). Second, although we adjusted for a variety of factors, including age, gender, education, marital status, place of residence, consumption level, smoking status, and drinking status, we were unable to correct for dietary habits, genetic factors, and so on, due to data limitations or other reasons. Third, the participants included in this study were all middle-aged and older Chinese people aged ≥45 years, and the applicability of the findings to other populations remains to be verified. In the future, larger prospective cohort studies are needed to further explore the relationship between METS-IR and UA and the risk of stroke, with the aim of providing a scientific basis for the prevention of stroke. Finally, a limitation of our study is the borderline significance of the nonlinear relationship between METS-IR and stroke risk (P = 0.047), which suggests caution in interpreting the result. While the analysis indicates nonlinearity, the proximity of the p-value to 0.05 raises the possibility of a more linear pattern that warrants further investigation.

## 5 Conclusion

In conclusion, we found that METS-IR and UA levels were positively associated with an increased risk of stroke onset, and that CRP mediated these relationships. The combined effect suggests that IR and hyperuricemic state may act together to significantly increase the risk of stroke in patients. Our study has important public health implications for the prevention of stroke events, suggesting that healthcare professionals need to consider patients’ METS-IR, urea, and CRP levels in order to adequately assess the risk of stroke and identify high-risk individuals in a timely manner. Meanwhile, our study also suggests that improving insulin sensitivity and regulating CRP and uric acid levels may be important for preventing the risk of stroke occurrence.

## Data Availability

The datasets presented in this study can be found in online repositories. The names of the repository/repositories and accession number(s) can be found below: https://charls.charlsdata.com/users/profile/index/zh-cn.html.
